# Evaluation of metal-oxide semiconductors for the photocatalytic degradation of chloroquine phosphate in real-world water matrices

**DOI:** 10.1038/s41598-026-56732-x

**Published:** 2026-06-16

**Authors:** Fangyuan Zheng, Roberto Fernández de Luis, Senentxu Lanceros-Méndez, Pedro M. Martins

**Affiliations:** 1 Centre of Molecular and Environmental Biology (CBMA), University of Minho, 4710-057 Braga, Portugal; 2 Institute for Research and Innovation on Bio-Sustainability (IB-S), University of Minho, 4710-057 Braga, Portugal; 3 BCMaterials, Basque Center for Materials, Applications and Nanostructures, UPV/EHU Science Park, 48940 Leioa, Spain; 4 Laboratory of Physics for Materials and Emergent Technologies, Physics Centre of Minho and Porto Universities (CF-UM-UP) and LaPMET, University of Minho, 4710-057 Braga, Portugal; 5 IKERBASQUE, Basque Foundation for Science, 48009 Bilbao, Spain

**Keywords:** Chloroquine phosphate, Photocatalysis, Reactive oxygen species, Semiconductor nanoparticles, Water matrices, Chemistry, Environmental sciences, Materials science, Nanoscience and technology

## Abstract

The persistence of pharmaceuticals in aquatic environments poses an increasing threat to aquatic ecosystems and human health, as conventional wastewater treatment plants often fail to remove contaminants of emerging concern (CECs) effectively. This work offers a systematic comparative assessment of the photocatalytic activity of TiO₂, ZnO, CeO₂, Bi₂O₃, and WO₃ nanoparticles for degrading chloroquine phosphate (CLQ), a widely used antiviral and anti-inflammatory drug, under ultraviolet irradiation in ultrapure water, drinking water, and synthetic seawater. The semiconductors were thoroughly characterised for morphology, crystalline structure, optical bandgap, and surface charge. Reactive oxygen species (ROS) generation, including hydroxyl radicals (^·^OH) and singlet oxygen (¹O₂), was measured to clarify degradation efficiency in each matrix. TiO₂ demonstrated the highest photocatalytic efficiency, achieving up to 83% CLQ degradation in drinking water, consistent with its superior ROS production. ZnO and CeO₂ showed moderate activity, with performance significantly influenced by pH and ionic composition, while Bi₂O₃ and WO₃ remained suppressed, especially in complex water matrices. Overall, this study emphasises the importance of assessing photocatalysts under realistic environmental conditions and identifies TiO₂ as the most robust and adaptable semiconductor for removing CLQ, even in complex water matrices.

**Supplementary Information:**

The online version contains supplementary material available at https://doi.org/10.1038/s41598-026-56732-x.

## Introduction

Water is essential for human life and ecosystems^1^. However, increasing water pollution leaves nearly 2 billion people without access to safe drinking water^2^. Moreover, the consumption of contaminated water causes over 1.5 million deaths per year, mainly due to diarrhoeal diseases^3^. In this context, awareness of the presence of contaminants of emerging concern (CECs), such as pharmaceuticals, in the aquatic environment is increasing due to their potential adverse effects on aquatic organisms and human health, despite their occurrence at very low concentrations (µg/L to ng/L)^4,5^.

Among these CECs, chloroquine phosphate (CLQ), a traditional anti-inflammatory and antiviral drug used for the treatment of malaria, rheumatoid arthritis, and systemic lupus erythematosus, has recently attracted significant attention^6^. It was recognised as an effective medicine against SARS-CoV-2 and was approved for COVID-19 treatment by both international and national organisations^7,8^, leading to widespread use and overproduction^9,10^. Due to its complex molecular structure and recalcitrant nature, CLQ is neither fully metabolised by the human body nor efficiently degraded by conventional wastewater treatment plants (WWTPs), leading to increased concentrations in the aquatic environments^8,11^. Furthermore, the concentration of CLQ in raw wastewater, secondary effluent and rivers was predicted to be 857 ng/L, 320 ng/L and 32 ng/L, respectively^12^. The release of CLQ into the environment may pose a potential risk to living organisms since it is associated with adverse effects in mammals, including blindness, cardiac arrest, and renal failure^13^. Nevertheless, there is limited work focused on CLQ removal. Therefore, developing effective water treatment methods to degrade this resilient compound is paramount.

Advanced oxidation processes (AOPs), which rely on the generation of highly oxidising species such as hydroxyl radical (^·^OH), have attracted considerable attention for degrading a wide range of organic pollutants, including CECs^14–18^. Among these methods, photocatalysis has attracted considerable attention for the degradation of CECs in water treatment^18–22^, as it can operate under ambient conditions and harness solar energy, offering a cost-effective and eco-friendly strategy for water remediation^23–25^. Typically, photocatalysis occurs when a catalyst absorbs a photon with energy (${h}_{\nu })$ equal to or higher than its bandgap (E_g_) under irradiation, promoting an electron (${e}_{CB}^{-}$) from its valence band (VB) to the conduction band (CB), creating a positive hole (${h}_{VB}^{+}$) in the valence band. These photogenerated electron-hole pairs will then react with H_2_O and O_2_ to produce the highly reactive oxygen species (ROS) such as hydroxyl radical (^·^OH), superoxide radical (^·^O_2_^−^) and singlet oxygen (^1^O_2_), which enable the degradation of pollutants into innocuous compounds^26,27^.

Several semiconductors have been extensively studied as photocatalysts for environmental remediation applications^18,28^. Among them, titanium dioxide (TiO_2_), with a bandgap of 3.0–3.2 eV, is the most extensively used photocatalyst due to its abundance, low cost, high stability, and remarkable photocatalytic activity for degrading various organic contaminants^24,29^. Zinc oxide (ZnO) has a similar bandgap. It exhibits good optoelectronic, piezoelectric, and photochemical properties^28,30^, while the oxygen vacancies on its surface can promote the generation of hydroxyl radicals (·OH) and improve the photocatalytic activity^30,31^. Cerium dioxide (CeO_2_), with a bandgap of 2.8–3.1 eV 32, also shows strong potential for photocatalytic applications^32,33^, as it exhibits low toxicity, thermal stability, chemical inertness, and the ability to undergo Ce^4+^/Ce^3+^ transformation, which facilitates the formation of oxygen vacancies and enhances photocatalytic efficiency^34,^^35^. In addition, research interest has shifted to emerging visible-light-responsive semiconductors such as bismuth oxide (Bi₂O₃) and tungsten trioxide (WO₃)^36,37^. Bi_2_O_3_, with a bandgap 2.4–2.8 eV^38^, is particularly attractive for environmental applications due to its non-toxic and biocompatible properties^37–39^. Similarly, WO₃ with a band gap of 2.4–2.8 eV^40^ is regarded as another emerging photocatalyst because of its low toxicity, high resistance to photocorrosion, and notable stability in acidic aqueous environments^40,41^. Nevertheless, comparative studies assessing the photocatalytic behaviour of these materials remain scarce, particularly for the removal of CECs from contaminated water. Therefore, a systematic evaluation of these conventional and emerging photocatalysts under identical experimental conditions is essential to understand the influence of semiconductor type on photocatalytic performance and to identify the most efficient candidate for water remediation. Further, most photocatalytic studies of CECs degradation have been conducted in ultrapure water, without considering the complex composition of natural water matrices^42,43^. Compared to ultrapure water, natural water matrices present different physicochemical parameters that can significantly affect the photocatalytic activity^42,43^. For instance, the presence of natural organic matter (NOM) and inorganic ions such as bicarbonate (HCO_3_^−^), chloride (Cl^−^), and sodium (Na^+^) can affect the adsorption of contaminants on the surface of the photocatalyst, reducing the number of active sites and competing for the generated ROS, thereby decreasing the final degradation efficiency^42,44^. Furthermore, pH variations in natural water can affect ROS generation and alter the photocatalyst surface charge, thereby enhancing or reducing photocatalytic activity^43,44^. Additionally, the conductivity of natural water affects the photocatalyst’s efficiency^43,45^. To date, only a limited number of works have explored the photocatalytic degradation using real water samples. Among them, many studies used only a single photocatalyst^43,44^, limiting our understanding of how different photocatalytic materials perform under realistic conditions. Other works evaluated the effect of natural water bodies on photocatalytic efficiency by studying individual ions separately in ultrapure water^46^^47^, which does not account for the simultaneous and realistic presence of various components in real water matrices, where synergistic interactions or inhibitory effects can significantly affect the overall performance of the photocatalytic process. In addition, drinking water is directly related to daily human consumption and public health, and several contaminants of emerging concern have already been detected in this type of water system, while the ocean is the main final receptor of many environmental pollutants^43^. Therefore, it is important to focus on the influence of such water matrices on the photocatalytic degradation.

Thus, the present work focuses on a comparative study of the photocatalytic activity of titanium dioxide, zinc oxide, cerium dioxide, bismuth oxide, and tungsten trioxide nanoparticles for degrading chloroquine phosphate in three representative water matrices: ultrapure water, drinking water, and synthetic seawater. In parallel, a systematic assessment of reactive oxygen species generation during the process is performed. This study provides comprehensive insights into the applicability of the selected photocatalysts under different water matrices. It contributes to the development of more effective water treatment methods for the degradation of contaminants of emerging concern in real-world scenarios.

## Materials and methods

### Materials

Titanium dioxide (TiO_2_, > 99.5%) nanoparticles were obtained from Evonik Industries AG. Zinc oxide (ZnO, > 97%), cerium (IV) oxide (CeO_2_, ≥ 99%), bismuth (III) oxide (Bi_2_O_3_, ≥ 99.8%) and tungsten (VI) oxide (WO_3_, ≥ 99%) nanoparticles, calcium chloride (CaCl_2_ 2H_2_O, ≥ 99%) and L-histidine (98%) were purchased from Sigma-Aldrich. Sodium hydroxide (NaOH, 98.0–100.5%) was supplied by Panreac. Hydrochloric Acid (HCl, 37%) was purchased from LABKEM. Terephthalic acid (TA, ≥ 99%) and 2-hydroxyterephthalic acid (2-HTA, ≥ 98%) were received from Thermo Fisher Scientific. N, N-dimethyl-4- nitrosoaniline (C_8_H_10_N_2_O, 98%) was provided by Alfa Aesar. Sodium chloride (NaCl, 99.5%), and sodium sulphate (Na_2_SO_4_, ≥ 99%) were supplied by PanReac AppliChem. Potassium chloride (KCl, Heavy metals ≤ 5 ppm), sodium bicarbonate (NaHCO_3_, 99.7–100.3%), and magnesium chloride (MgCl_2_·6H_2_O, ≥ 99%) were received from Fisher Bioreagents. Potassium bromide (KBr, ≥ 99%), sodium fluoride (NaF, 99%), and strontium chloride (SrCl_2_·6H_2_O) were supplied through Thermoscientific. Boric acid (H_3_BO_3_, ≥ 99.8%) was provided by Fisher Chemical. Milli-Q ultrapure water (resistivity 18.2 MΩ·cm) was used in all experiments unless otherwise specified. Commercial drinking water (see main features in Table S1) was obtained from Aguas das Celdas de Penacova, S.A. (https://www.caldasdepenacova.pt/home-2-1). Synthetic seawater (see main features in Table S2) was prepared according to a previously developed protocol^48^. Chloroquine phosphate (CLQ, Pharmaceutical Secondary Standard; Certified Reference Material, C_18_H_26_CIN_3_·2H_3_PO_4_) with maximum spectral absorption at a wavelength of 342 nm was purchased from Sigma-Aldrich.

### Characterisation techniques

Transmission electron microscopy (TEM) images were obtained using a JEOL JEM 1400 Plus microscope operating at 100 kV in a bright field. The analysis of the micrographs was performed using the ImageJ software package. For sample preparation, the nanoparticle powder was dispersed in ultrapure water and sonicated for 1 min. Afterwards, 2µL of the suspension was placed on a 400-mesh carbon-coated copper grid and dried at room temperature. The particle size distribution was obtained after analysing the diameter of at least 100 nanoparticles employing the ImageJ software package (version 1.54).

The purity and the crystal phase of the nanoparticles were analysed by X-ray diffraction (XRD) using a Philips X’Pert PRO automatic diffractometer operating at 40 kV and 40 mA. The equipment has a theta-theta configuration, a secondary monochromator with Cu-Kα radiation (λ = 1.5418 Å) and a PIXcel solid-state detector (active length in 2θ 3.347°). Data were collected over a 2θ range of 5–80° with a step size of 0.026°, and an acquisition time per step of 60 s at room temperature. 1° fixed Soller slit and divergence slit, giving a constant sample illumination volume, were used. The size of the crystalline domains the nanoparticles was determined by the Scherrer Eq. (1)^49^:

1 $$D=\frac{k\lambda }{\beta cos\theta }$$

Where D (nm) is the size of the crystallite domains, k is the shape factor with a value of 0.9, l = 1.5418 Å is the wavelength of the X-rays, β is the full width at half maximum (FWHM, radians) obtained from the most intense peak after subtracting the instrumental contribution (0.1 radians), and θ is the Bragg angle (degrees).

Diffuse reflectance spectroscopy (DRS) measurements were conducted in the 200–2200 nm wavelength range using a UV-Visible-NIR Jasco V-770 spectrometer equipped with a 150 mm diameter integrating sphere coated with Spectralon with 1 nm spectral resolution. A Spectralon reference was used to measure 100% reflectance, while internal attenuators were used to determine zero reflectance, thereby removing background and noise. The sample was placed in a quartz cuvette, sealed, and mounted on a Teflon sample holder for the DRS measurement. The reflectance spectra were subsequently converted to Kubelka–Munk (K–M) absorption factors to evaluate the absorption spectra of the powders by using the K–M Eq. (2)^50^:

2 $$F\left(R\right)={(1-{R}_{\infty })}^{2}/\left(2{R}_{\infty }\right)$$

where F(R) corresponds to absorbance and R_∞_ (R_Sample_/R_BaSO4_) is the sample’s reflectance. The bandgap of the sample was estimated using the Tauc plot Eq. (3):

3 $${\left[F\left(R\right)h \upsilon \right]}^{1/n} versus h \upsilon$$

where *h* is Planck’s constant, ʋ the frequency, and n is the sample transition parameter taken as *n* = 2 for indirect transition^51^, as previously reported for several photocatalysts such as TiO_2_^24^. ZnO^27^, CeO_2_^52^, Bi_2_O_3_ eV^38^ and WO_3_^53^ nanoparticles.

Dynamic light scattering (DLS) and zeta-potential measurements were carried out using a Zetasizer NANO ZS-ZEN3600 (Malvern Instruments Limited, United Kingdom), equipped with a He–Ne laser (wavelength 633 nm) and backscatter detection (173°). The nanoparticles were dispersed at 1 mg/mL in ultrapure water and sonicated at room temperature for 1 h to prevent aggregation. For DLS assays, each sample was measured at pH = 11 to obtain the hydrodynamic diameter. Regarding the zeta-potential assays, measurements were performed after adjusting the pH of the samples to 3, 5, 7, 9, and 11 using HCl (0.1 M) and NaOH (0.1 M) solutions. All measurements for DLS and zeta potential were performed five times to ensure the accuracy and repeatability of the results. The results were obtained using the Smoluchowski model^54^. Zetasizer 7.13 software was used to estimate the hydrodynamic diameter of the nanoparticles (Z-average), the polydispersity index (PDI), and the zeta potential.

### Photogenerated ROS (^·^OH and ^1^ O_2_) measurements

#### Detection and quantification of hydroxyl radicals (^·^OH)

Based on a previously reported protocol^27^, the hydroxyl radicals (^·^OH) were detected and quantified by the hydroxylation reaction of terephthalic acid (TA) to 2-hydroxyterephthalic acid (2-HTA) (Figure S1).

To this end, TA was dissolved in a NaOH ultrapure water solution (2 mM) to achieve a concentration of 0.5 mM. Afterwards, 50 mg of nanoparticles were dispersed in 50 mL of the TA solution, and the mixture was stirred in the dark for 30 min. Subsequently, the suspension was irradiated under UV illumination while stirring continuously. 1 mL aliquot was withdrawn at the beginning and end of the irradiation period and centrifuged to allow the nanoparticles to settle. In the following, 200 µL of supernatant was collected, and its luminescence was measured three times using an Infinite 200 Pro microplate reader. Emission from 2-HTA was measured at its maximum of 425 nm with an excitation wavelength of 315 nm^55^ in the fluorescence spectrum. A standard calibration curve for 2-HTA was established in parallel to correlate the fluorescence signal and the concentration of the hydroxyl radicals. Further, the hydroxyl radicals (^·^OH) detection and quantification experiments were repeated employing commercial drinking water and synthetic seawater as water matrices.

#### Detection and quantification of singlet oxygen (^1^O_2_)

Singlet oxygen (^1^O_2_) detection and quantification were done following the histidine test (Figure S2)-experimental protocol described in a previous study^27^.

In brief, 40 mL of L-histidine solution (0.2 mM) and 10 mL of N, N-pnitrosodimethylaniline solution (0.2 mM) were initially prepared using ultrapure water and then mixed. Afterwards, 50 mg of nanoparticles were added to the solution and stirred in the dark for 30 min. Later, this suspension was illuminated under UV radiation while maintaining the agitation for 60 min. 1 mL aliquot was withdrawn at the beginning and end of the irradiation period, centrifuged, and its UV-Vis absorbance at 440 nm was measured in an Infinite 200 Pro microplate reader. In parallel, a standard calibration curve was established to correlate the UV-Vis absorbance values and the concentration of N, N-dimethyl-4-nitrosoaniline. Additionally, the singlet oxygen (^1^O_2_) detection and quantification assays were repeated using commercial drinking water and synthetic seawater as water matrices.

### Photocatalytic activity

The nanoparticles’ photocatalytic activity was evaluated in different water matrices under UV illumination. Initially, a CLQ solution at 30 mg/L was prepared separately in ultrapure water, commercial drinking water, and synthetic seawater. This concentration was selected based on the limitations of detecting the pollutant with the UV-Vis spectrophotometer and the potential for monitoring its degradation. Afterwards, 50 mg of nanoparticles as photocatalysts were added and stirred in 50 mL of the CLQ solution (30 mg/L) for 30 min in the dark to achieve the adsorption-desorption equilibrium before initiating the photocatalytic tests.

The UV degradation assays were performed in a photoreactor equipped with 8 W-UV lamps with an emission peak at 365 nm for 60 min. The suspensions of photocatalysts and CLQ-solutions were stirred in a 100 mL beaker under UV illumination. The distance between the solution and the lamp was 13.5 cm, and the irradiance at the sample was 3.3 W/m^2^. 1 mL aliquot was withdrawn at the beginning and end of the assays, centrifuged to remove the photocatalysts, and then 200 µL of the supernatant was collected, and its UV-Vis spectra were performed three replicates for each measurement using a microplate reader Infinite 200 Pro. The CLQ concentration in the solution was followed by recording the absorbance at λ_max_ = 342 nm. The photocatalytic degradation efficiency (DE) was determined by Eq. (4):

4 $$\mathrm{DE}=\left(\frac{{A}_{0}-A}{{A}_{0}}\right) \times 100\%$$

where A_0_ and A represent the absorbance at λ_max_ = 342 nm at time t = 0 and at a given time, respectively^56^.

## Results and discussion

### Nanoparticle characterisation

**Fig. 1:**
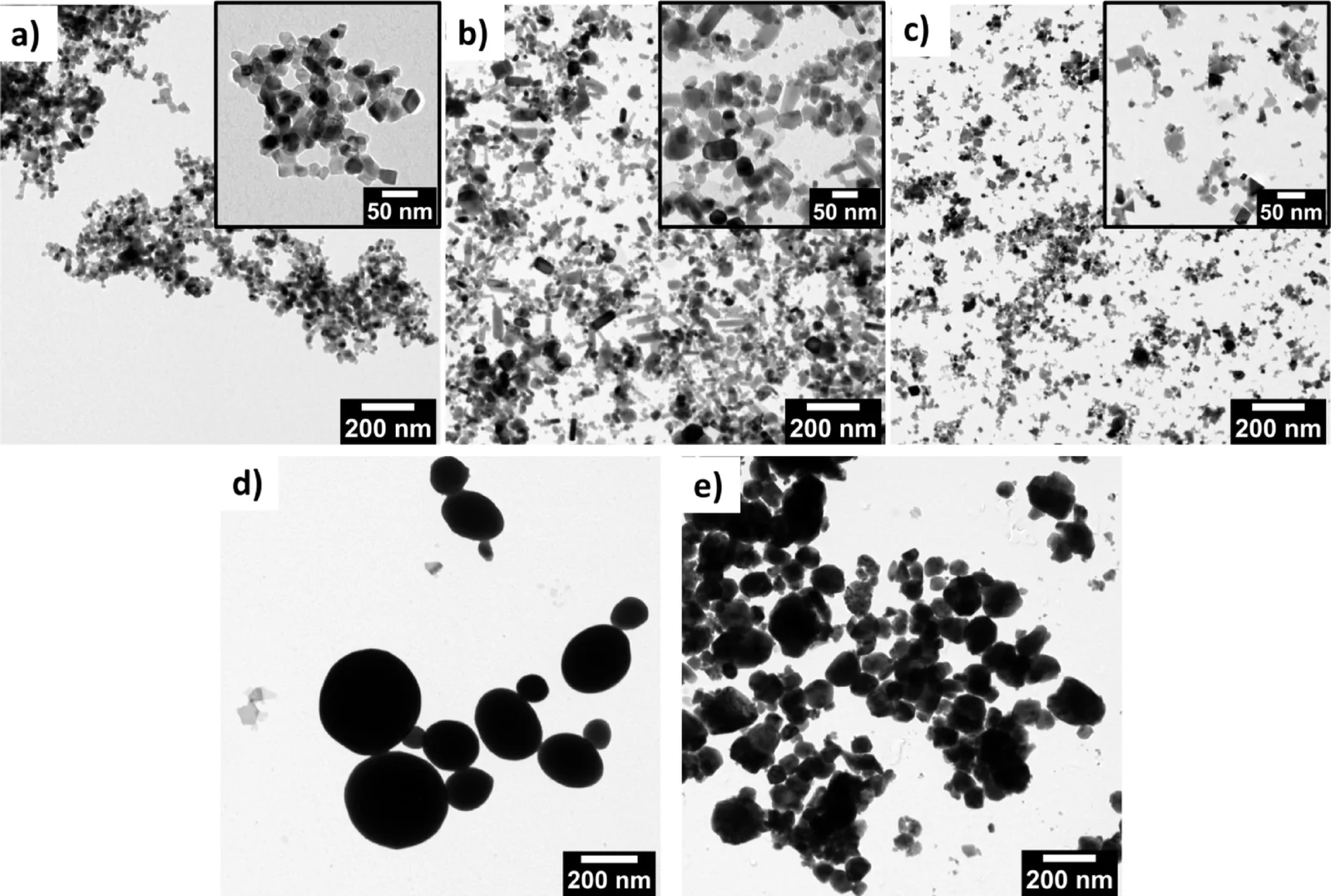
TEM images of TiO_2_ (**a**), ZnO (**b**), CeO_2_ (**c**), Bi_2_O_3_ (**d**) and WO_3_ (**e**) nanoparticles.

The morphology of TiO_2_, ZnO, CeO_2_, Bi_2_O_3_, and WO_3_ nanoparticles was evaluated using TEM, and their diameters were quantified using ImageJ software. Figure 1 and Figure S3 present the TEM images and size distribution of the samples, respectively, and Table 1 summarises the determined average particle size.

TiO₂ nanoparticles exhibit a spherical morphology with a uniform size distribution, having an average particle diameter of 27.9 ± 6.5 nm (Fig. 1a). Figure 1b) identifies both nanorod and spherical shapes for ZnO nanoparticles, with a size distribution ranging from 10 to 80 nm and an average particle diameter of 34.6 ± 13.5 nm. Moreover, CeO_2_ is a more heterogeneous sample (Fig. 1c), presenting nanoparticles with morphologies ranging from spherical to square and triangular. The diameter of CeO_2_ nanoparticles ranges from 6 to 40 nm, with an average of 18.1 ± 7.8 nm. In terms of Bi_2_O_3_ nanoparticles (Fig. 1d), a spherical shape is clearly identified in the micrographs, with a size distribution between 50 and 500 nm and an average particle diameter of 211.49 ± 92.74 nm. Similarly, as shown in Fig. 1e), WO_3_ nanoparticles Fig. 1e) are nearly spherical, with a size distribution varying from 50 to 250 nm and an average particle diameter of 113.97 ± 38.27 nm.

**Fig. 2:**
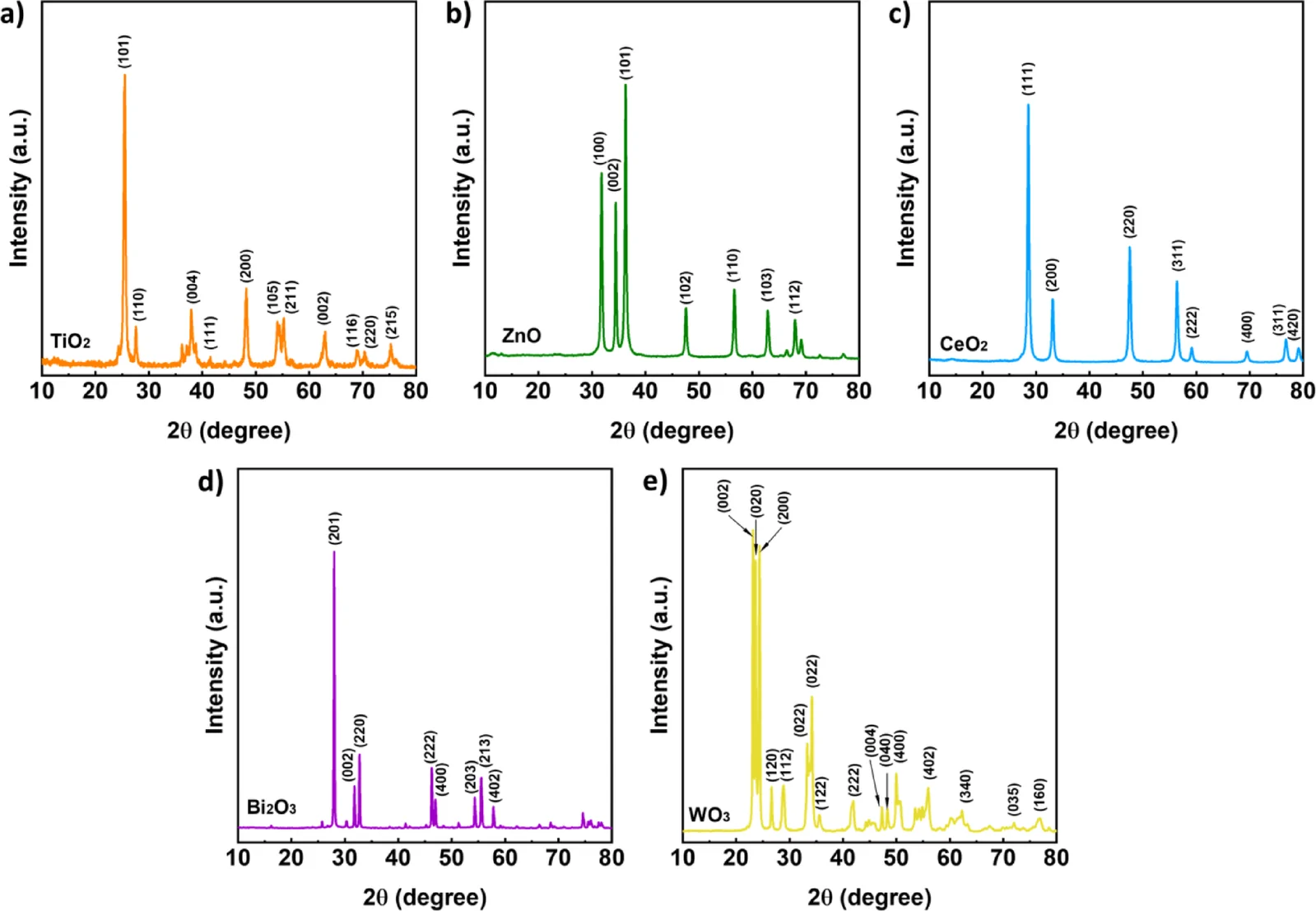
X-ray diffraction spectra of TiO_2_ (**a**), ZnO (**b**), CeO_2_ (**c**), Bi_2_O_3_ (**d**) and WO_3_ (**e**) nanoparticles.

XRD analysis was performed to identify the main crystalline phase of each sample. the nanoparticles’ crystalline phase and determine their crystallite size. Further, the Scherrer analysis enabled determining the size of their crystalline domains. Figure 2 illustrates the XRD patterns of each nanoparticle, showing the Bragg reflections corresponding to the assigned crystalline phases. In detail, TiO_2_ nanoparticles (Fig. 2a) are composed of a mixture of TiO_2_ anatase and rutile phases, in good agreement with the literature for a TiO_2_-P25 literature^27,57^. ZnO nanoparticles (Fig. 2b) display the characteristic diffraction peaks corresponding to the pure hexagonal wurtzite phase^58^, aligned with the literature protocol^27,59^. Figure 2c) confirms the cubic fluorite structure of CeO_2_ nanoparticles, in line with the literature^52,60^.

Additionally, Fig. 2d) identifies the phase beta (tetragonal) crystal structure of Bi_2_O_3_ nanoparticles^61,62^. WO_3_ nanoparticles (Fig. 2e) show a monoclinic crystalline structure, which is in line with the literature^63,64^. Notably, even though the nanoparticles are small, the XRD patterns exhibit sharp, intense diffraction peaks, indicating well-preserved long-range structural ordering.

The size of the crystalline domains of the samples, calculated by the Scherrer equation (Eq. 1), is listed in Table 1. These values are slightly smaller than the TEM sizes, since they indicate the average crystallite size of the samples, ot the nanoparticle size^49^. The sizes of the crystalline domains for TiO_2_ (26.43 nm), ZnO (33.73 nm), and CeO_2_ (17.38 nm) are very close to those obtained by microscopy, an agreement that indirectly confirms that each nanoparticle is a single crystallite of the metal-oxide photocatalysts. Instead, the size of the crystalline domains of Bi_2_O_3_ (100.50 nm) and WO_3_ (70.48 nm) - are approximately half of the average particle diameter by TEM. This finding indicates that the Bi_2_O_3_ and WO_3_ nanoparticles are composed of multiple domains with different crystalline orientations.

**Fig. 3:**
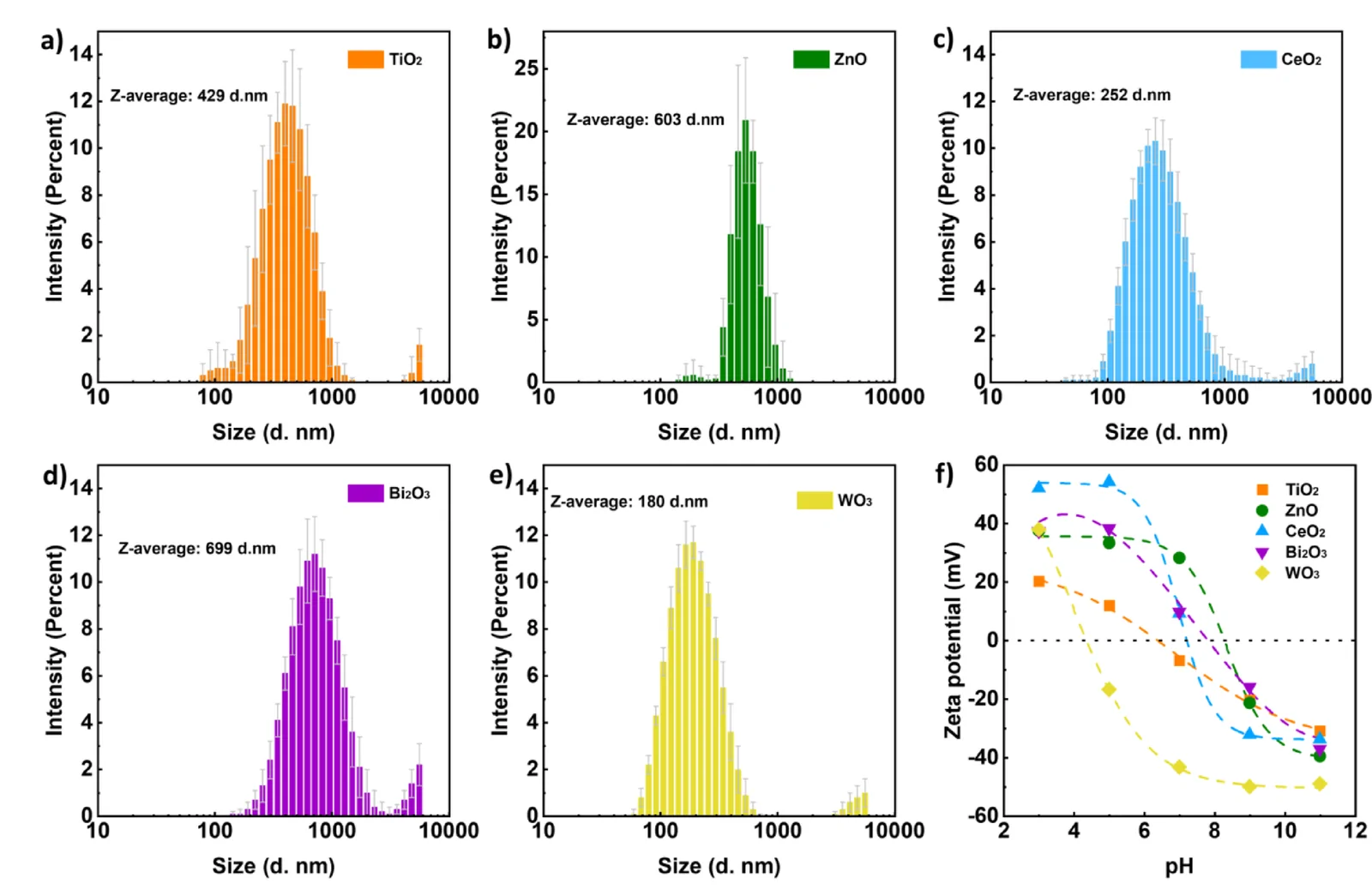
Intensity of the hydrodynamic size distribution and respective Z-average value for TiO_2_ (**a**), ZnO (**b**), CeO_2_ (**c**), Bi_2_O_3_ (**d**) and WO_3_ (**e**) nanoparticles and zeta potential measurements (**f**) performed at different pHs (3, 5, 7, 9, and 11).

The hydrodynamic size distribution histograms (Fig. 3a–e), along with the estimated average sizes (Z-average) and the polydispersity index (PDI) values (Table 1) for all the samples, were determined by DLS measurements at pH 11. For TiO_2_ nanoparticles (Fig. 3a), the hydrodynamic diameter is 429 ± 18 nm, with a PDI of 0.378 ± 0.010. ZnO nanoparticles (Fig. 3b) present a hydrodynamic size of 603 ± 19 nm, with a PDI of 0.267 ± 0.057. Furthermore, CeO_2_ nanoparticles (Fig. 3c) show a diameter of 252 ± 3 nm and a PDI of 0.250 ± 0.014. For Bi_2_O_3_ nanoparticles (Fig. 3d), the hydrodynamic diameter is 699 ± 23 nm with a PDI of 0.299 ± 0.031. Figure 3e) shows a hydrodynamic size of 180 ± 3 nm and a PDI of 0.226 ± 0.027 for WO_3_ nanoparticles. Among all samples, Bi_2_O_3_ nanoparticles possess the largest hydrodynamic size, whereas WO_3_ nanoparticles possess the smallest. Notably, the particle diameters obtained by DLS were significantly larger than those determined by TEM, which can be attributed to the formation of nanoparticle aggregates in aqueous media, leading to an increase in size during DLS analysis^24,29^. On the other hand, the relatively high PDI values of all samples also indicate their polydispersity in aqueous media, implying a wide particle size distribution due to agglomeration^65,66^. Although TiO_2_ nanoparticles show a homogeneous particle size distribution in TEM measurements, they exhibit the highest PDI value (≈ 0.378) in DLS analysis, implying a greater tendency to form agglomerates in aqueous medium compared to the others. Under the same conditions, WO_3_ nanoparticles display the lowest PDI value (≈ 0.226), indicating a relatively narrow hydrodynamic size distribution among all samples.

**Table 1:** Summary of the nanoparticle sizes (nm) determined by TEM, XRD and DLS measurements and the polydispersity index (PDI) estimated by DLS measurements.

Sample	TEM	XRD	DLS	
	Particle size (nm)	Crystalline size (nm)	Hydrodynamic size (nm)	PDI
TiO_2_	27.9 ± 6.5	26.43	429 ± 18	0.378 ± 0.095
ZnO	34.6 ± 13.5	33.73	603 ± 19	0.267 ± 0.057
CeO_2_	18.1 ± 7.8	17.38	252 ± 3	0.25 ± 0.014
Bi_2_O_3_	211.5 ± 92.7	100.5	699 ± 23	0.299 ± 0.031
WO_3_	113.9 ± 38.3	70.48	180 ± 3	0.226 ± 0.027

The surface charge of the nanoparticles dispersed in ultrapure water was studied by zeta-potential measurement in a pH range from 3 to 11 (Fig. 3f) to confirm their stability under different conditions. When the absolute zeta potential exceeds 30 mV, nanoparticles are considered electrostatically stable due to enhanced repulsion between particles, which can prevent agglomeration^67,68^.

In general, all samples, except WO_3_ nanoparticles, showed positive zeta potentials at acidic pH and negative zeta potentials at basic pH. Among them, TiO_2_ nanoparticles showed a zeta-potential modulus around 30 mV at pH 11, indicating that the TiO_2_ nanoparticles are more stable in alkaline conditions. ZnO nanoparticles exhibit high stability at pH values below 5 and above 11, where their zeta-potential values ranged between |30–40| mV. Moreover, CeO_2_ nanoparticles showed zeta-potential values around |50| mV at pH below 5 and around |30| mV at pH above 9, confirming their superior electrostatic stability^67,68^ at both acidic and basic pH. Bi_2_O_3_ nanoparticles exhibit a zeta potential of approximately 40 mV at pH values below 5 and above 11, indicating good stability in both acidic and highly alkaline conditions. WO_3_ nanoparticles possess the Z-potential values between |40–50| mV when pH was below 3 and above 7, implying that the WO_3_ nanoparticles show stability in strong acid, neutral and basic conditions. At pH 11, where the DLS measurements were performed, WO_3_ nanoparticles possessed the largest value |≈ 50| mV among all samples. Higher zeta potential values indicate a higher surface charge, which enhances particle repulsion, improving stability and preventing agglomeration^67,68^. This confirms the DLS results, which showed that stronger repulsions between particles can prevent aggregation and lead to a smaller hydrodynamic size and a relatively narrow size distribution.

Another different behaviour among nanoparticles is observed at the point of zero charge (PZC), where the particles’ surface charge is equal to zero^69^. Table 2 presents the PZC of each type of nanoparticles. TiO_2_ and CeO_2_ nanoparticles exhibit a PZC of 69 at pH neutral, between 6 and 7. Moreover, ZnO and Bi_2_O_3_ nanoparticles exhibit a PZC around pH 8. In contrast, the PZC of WO_3_ is shifted to around 4 at acidic pH.

**Table 2:** Point of zero charge (PZC) in ultrapure water of the different nanoparticles.

Sample	Point of zero charge (PZC)
TiO_2_	6.37 ± 0.06
ZnO	8.28 ± 0.04
CeO_2_	7.21 ± 0.04
Bi_2_O_3_	7.80 ± 0.08
WO_3_	4.30 ± 0.03

**Fig. 4:**
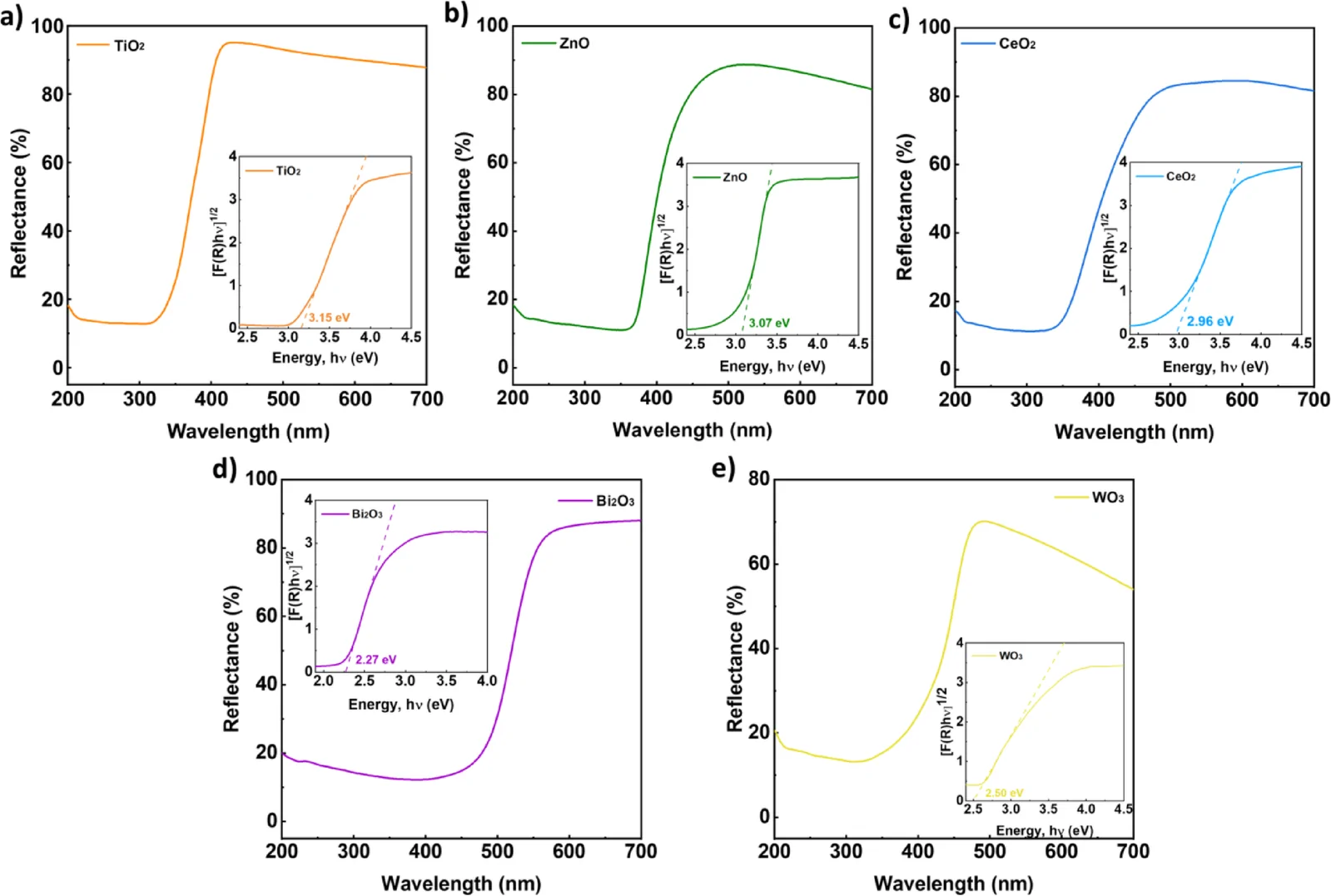
UV-Vis reflectance spectra of TiO_2_ (**a**), ZnO (**b**), CeO_2_ (**c**), Bi_2_O_3_ (**d**) and WO_3_ (**e**) nanoparticles with the estimation of the bandgap (inset) at [F(R)hʋ]^1/2^ → 0.

Moreover, the optical properties of the samples were studied by DRS, as shown in Fig. 4. In the UV range (200–400 nm), all nanoparticles exhibit very low reflectance, indicating strong UV absorption. In particular, reflectance around 365 nm, the UV irradiation wavelength used in this study, is 40%, 11%, 22%, 12% and 17% for TiO₂, ZnO, CeO₂, Bi_2_O_3_ and WO₃, respectively. This confirms efficient absorption of the incident UV light under the experimental conditions. Nonetheless, these materials displayed distinct reflectance behaviours in the visible range (400–700 nm). TiO_2_ nanoparticles (Fig. 4a) reflect nearly 90% of the visible radiation. In contrast, ZnO and CeO_2_ (Fig. 4b and c) nanoparticles reflect approximately 80%. Interestingly, Bi_2_O_3_ nanoparticles (Fig. 4d) present a very low reflectance between 400 and 550 nm and show a very high reflectance ≈ 88% from 550 to 700 nm, implying that Bi_2_O_3_ nanoparticles can absorb part of the visible radiation. Additionally, WO_3_ nanoparticles (Fig. 4e)) exhibit the lowest reflectance among all the samples in the visible range, with a maximum of 54%, presenting an excellent visible light absorption capacity.

The bandgap of the nanoparticles was calculated from the DRS spectrum using Eqs. (2) and (3), as shown in the inset graph of Fig. 4. The TiO_2_ nanoparticles present a bandgap of 3.15 ± 0.03 eV, typical for TiO_2_ (3.0–3.2 eV)^70^. The ZnO and CeO_2_ nanoparticles display lower band gaps than TiO_2_, with values of 3.07 ± 0.09 eV and 2.96 ± 0.06 eV, respectively. On the other hand, significant bandgap reductions are observed in Bi_2_O_3_ and WO_3_ nanoparticles, being 2.27 ± 0.02 eV and 2.50 ± 0.06 eV, respectively. These values confirm their capacity to harvest visible light. Among all samples, TiO_2_ nanoparticles show the largest bandgap, while Bi_2_O_3_ nanoparticles possess the smallest one.

### Photogenerated ROS (^·^OH and ^1^ O_2_) measurements

Based on the estimated bandgap, the 365 nm UV radiation (~ 3.4 eV) employed in this work provided sufficient energy to excite the evaluated nanoparticles. Hence, experiments were performed to detect and quantify ROS. Reactive oxygen species (ROS) generated on the photocatalysts surface play an essential role in contaminant degradation via photocatalysis^26,27^. Additionally, the presence of different chemical species and variation in water pH significantly affect ROS generation^23,43^. Therefore, detecting and quantifying ROS during the photocatalytic process is crucial for a deeper understanding of nanoparticles’ photocatalytic activity and performance in different water matrices.

The following equations can express the ROS generation process of the photocatalyst after light irradiation^27,71^:

5 $$Semiconductor\underrightarrow{{h}_{\upsilon }}{h}_{VB}^{+}+{e}_{CB}^{-}$$

6 $${H}_{2}O+{h}_{VB}^{+}\to { }^{{\cdot }}OH+ {H}^{+}$$

7 $${O}_{2}+{e}_{CB}^{-}\to {{ }^{{\cdot }}O}_{2}^{-}$$

8 $${{ }^{{\cdot }}O}_{2}^{-}+{h}_{VB}^{+}\to { }^{1}{O}_{2}$$

As previously mentioned, ^·^OH radicals were detected via TA hydroxylation, while singlet oxygen (¹O₂) was measured by histidine assay.

**Fig. 5:**
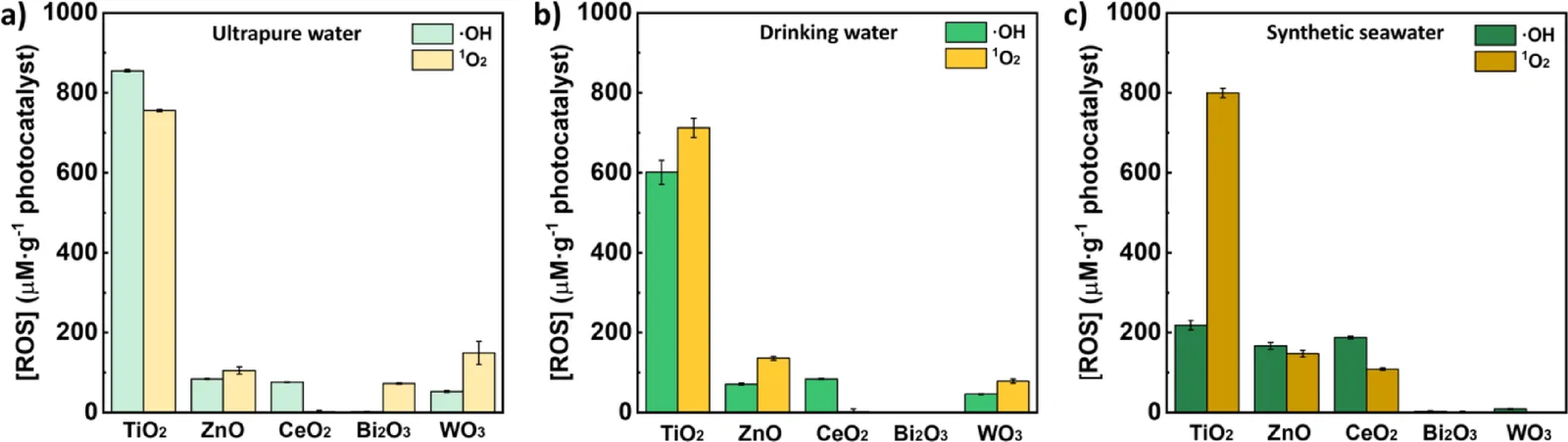
Quantification of photogenerated hydroxyl radical (^·^OH) and singlet oxygen (^1^O_2_) by TiO_2_, ZnO, CeO_2_, Bi_2_O_3_ and WO_3_ nanoparticles in ultrapure water (**a**), drinking water (**b**) and synthetic seawater (**c**) under 60 min of UV radiation.

Figure 5; Table 3 present the total amount of ROS generated by the different nanoparticles after 60 min of UV irradiation in ultrapure water, drinking water, and synthetic seawater. Firstly, the photocatalysts’ performance was analysed and compared under the same water matrix. Based on the reflectance values at 365 nm, the light absorption capability of the photocatalyst followed the order: ZnO > Bi₂O₃ > WO₃ > CeO₂ > TiO₂. However, ROS-generation behaviour did not follow the same trend, suggesting that light absorption efficiency alone does not determine the ROS generation capability.

- Ultrapure water

In ultrapure water (Fig. 5a), the assessed nanoparticles demonstrated different ROS generation capacity, following the order: TiO_2_ > WO_3_ > ZnO > CeO_2_ > Bi_2_O_3_. Among all the samples, TiO_2_ produced the highest levels of hydroxyl radicals (^·^OH; 855 µM·g^− 1^) and singlet oxygen (¹O₂; 756 µM·g^− 1^), making it the most active nanoparticle. Although WO_3_ was the second most active photocatalyst, its activity was significantly lower, generating only 6% of the ^·^OH and 20% of the ¹O₂ produced by TiO₂. Furthermore, ZnO demonstrated performance comparable to WO_3_ but with a slight reduction. CeO_2_ and Bi_2_O_3_ produced the lowest amounts of ROS among all the nanoparticles, with 76 µM·g^− 1^ of ^·^OH and 72 µM·g^− 1^ of ¹O₂, respectively.

- Drinking water

In drinking water (Fig. 5b), the ROS generation behaviour was very similar to that observed in ultrapure water, except for ZnO and WO_3_, following the order: TiO_2_ > ZnO > WO_3_ > CeO_2_ > Bi_2_O_3_. TiO₂ remained the most efficient nanoparticle, generating 601 µM·g^− 1^ of ^·^OH and 712 µM·g⁻¹ of ¹O₂, followed by ZnO with 71 µM·g^− 1^ of ^·^OH and 136 µM·g⁻¹ of ¹O₂. Moreover, WO_3_ showed approximately 40% lower efficiency than ZnO. CeO_2_ continued to show a low ^·^OH production, while Bi_2_O_3_ showed a negligible ROS production.

- Synthetic seawater.

In synthetic seawater (Fig. 5c), a different ROS generation trend of the nanoparticles was observed compared to ultrapure and drinking water, resulting in the order: TiO_2_ > ZnO > CeO_2_ > WO_3_ > Bi_2_O_3_. TiO_2_ maintained the highest activity, producing 219 µM·g^− 1^ of ^·^OH and 800 µM·g⁻¹ of ¹O₂. ZnO showed less efficiency than TiO_2_, generating 167 µM·g^− 1^ of ^·^OH and 147 µM·g⁻¹ of ¹O₂. Interestingly, CeO_2_ produced both ^·^OH and ¹O₂ with a performance like ZnO. In contrast, WO_3_ and Bi_2_O_3_ exhibited a negligible activity.

In general, across all samples, TiO_2_ showed the highest ROS generation activity in different water matrices, while Bi_2_O_3_ showed the lowest. ZnO and CeO_2_ nanoparticles demonstrated increased ROS generation with more complex compositions in the water matrix, whilst others decreased. WO_3_ lost its activity in synthetic seawater, whereas Bi_2_O_3_ was only active in ultrapure water. Overall, the type and complexity of the water matrix resulted in distinct effects on each nanoparticle.

To further understand the influence of the water matrix on ROS generation, a comparative analysis was carried out on ^·^OH and ^1^ O_2_ generation by the same nanoparticle in different water matrices. Regarding ^·^OH generation (Figure S4 a)), the increased complexity of the water matrix reduced the efficiency of TiO_2_, resulting in a 30% decrease in drinking water and up to 75% in synthetic seawater compared to ultrapure water. This reduced activity of TiO_2_ in drinking water can be attributed to the acidic condition (pH = 5.3) of drinking water, which was not favourable for ^·^OH generation due to a high amount of H^+^ ions^72^. Moreover, the positively charged surface of TiO_2_ in drinking water promoted the adsorption of inorganic anions such as HCO_3_^−^ and Cl^−^ and SO_4_^2−^, thereby reducing the number of available active sites for ^·^OH radical generation^73^. In addition, these anions can react with photogenerated ^·^OH and act as hole (${h}_{VB}^{+}$) scavengers, suppressing ^·^OH generation^72,74^. Although the basic condition (pH = 8.2) in synthetic seawater could favour the formation of ^·^OH radical^75^, TiO_2_ surface was negatively charged, which could repel the OH^−^ ions and simultaneously enhance the adsorption of inorganic cations such as Na^+^, Mg^2+^, and Ca^2+^, which further locked the active site and led to the efficiency loss^76^. Furthermore, the high ion concentration in synthetic seawater increased the ionic strength and conductivity of the medium, thereby promoting electron-hole recombination, yielding low ^·^OH generation^45,76^. Similarly, WO_3_ also presented a 13% efficiency loss of on ^·^OH generation in drinking water and was completely deactivated in synthetic seawater. This behaviour can be explained by the mechanisms previously described for TiO_2_. In both drinking water and synthetic seawater, WO_3_ surface was negatively charged, which enhanced the adsorption of cations and inhibited the attraction of OH^−^ ions, thereby decreasing the production of ^·^OH radical^76^. Additionally, the high ion concentration in synthetic seawater further enhanced electron-hole recombination, leading to the complete deactivation of WO_3_^45,76^. Bi_2_O_3_ showed a negligible activity under all conditions. In contrast, ZnO and CeO_2_ maintained the efficiency from ultrapure water to drinking water, while generating approximately twice the amount of ^·^OH radical in synthetic seawater compared to other water matrices. This stability in efficiency can be explained by the highly positively charged surface of ZnO and CeO_2_ in drinking water, which facilitated the efficient interaction with OH^−^ ions in the medium^72^ compensating for the loss of active sites caused by the adsorption of anions and maintaining ^·^OH generation. Moreover, the increased efficiency of ^·^OH generation in the synthetic sweater can be attributed to the larger number of OH^−^ ions in the basic condition^75^ and chemical structure of ZnO and CeO_2_. Although ZnO surface tends to zero charge in synthetic seawater, which minimised electrostatic attraction of both OH⁻ ions and other anions, the presence of oxygen vacancies on the ZnO surface favoured OH⁻ ions and water adsorption^30,77^ and prevented charge recombination^78^, thereby enhancing ^·^OH radical production. Similarly, the Ce⁴⁺/Ce³⁺ redox cycle of CeO_2_ also favoured the generation of oxygen vacancies on the surface, leading to the high production of ^·^OH radical^32,33,79^.

For ¹O₂ generation (Figure S4 b)), Bi₂O₃ was only active in ultrapure water, while WO₃ exhibited a 50% decrease in efficiency from ultrapure to drinking water and was completely deactivated in synthetic seawater. This efficiency reduction of Bi_2_O_3_ and WO_3_ in both drinking water and synthetic seawater was attributed to the increased ion concentration in the water matrix, which blocked the nanoparticles’ active sites^73^ and increased electron-hole recombination due to increased ionic strength and conductivity^45,76^, as previously mentioned in ^·^OH radical production. In addition, the highly negatively charged surface of WO_3_ could repulse the negatively charged ^·^O_2_^−^, hindering the production of ^1^O_2_^80,81^. In contrast, CeO_2_ only produced ¹O₂ in synthetic seawater since the basic condition of synthetic seawater promoted the Ce^4+^/Ce^3+^ redox cycle of CeO_2_ under illumination^82,83^, which facilitated the formation of oxygen vacancies^32,79^, enhancing O₂ adsorption and ¹O₂ generation^84^. Furthermore, TiO_2_ maintained ¹O₂ production efficiency across all water matrices. This behaviour could be attributed to the stabilisation of the intermediate superoxide radical (^·^O_2_^−^) at the TiO₂ surface within the pH range of 5–11^85^, which corresponds to the typical pH of these water matrices, thereby leading to consistent ¹O₂ formation regardless of the water type. Similarly, ZnO also preserved its ¹O₂ production efficiency across all water matrices. The stability of the efficiency of ZnO could be explained by the presence of oxygen vacancy that favoured the adsorption of dissolved O_2_ in water, improving the superoxide radicals (^·^O_2_^−^) generation^84^. Furthermore, the positively charged ZnO surface in drinking water and synthetic seawater also increased the electrostatic attraction with negatively charged species ^·^O_2_^−^, enhancing the subsequent production of ¹O₂^84^. Consequently, this synergistic effect maintained ¹O₂ generation by ZnO despite the complexity of the ions in the water matrix.

**Table 3:** Photogenerated hydroxyl radical (^·^OH) and singlet oxygen (^1^O_2_) by TiO_2_, ZnO, CeO_2_, Bi_2_O_3_ and WO_3_ nanoparticles in ultrapure water, drinking water and synthetic seawater under 60 min of UV radiation.

Samples	Photogenerated ROS (μM·g^-1^ photocatalyst)					
	Ultrapure water		Drinking water		Synthetic seawater	
	[^·^OH]	[^1^O_2_]	[^·^OH]	[^1^O_2_]	[^·^OH]	[^1^O_2_]
TiO_2_	855 ± 3	756 ± 3	601 ± 30	712 ± 24	219 ± 12	800 ± 12
ZnO	84 ± 1	106 ± 9	71 ± 2	136 ± 5	167 ± 8	147 ± 8
CeO_2_	76 ± 1	3 ± 3	84 ± 2	2 ± 7	188 ± 0	109 ± 3
Bi_2_O_3_	3 ± 0	72 ± 1	1 ± 0	0 ± 3	3 ± 0	1 ± 3
WO_3_	53 ± 1	149 ± 29	46 ± 0	78 ± 5	9 ± 0	0 ± 2

### Photocatalytic degradation of chloroquine phosphate under UV radiation

**Fig. 6:**
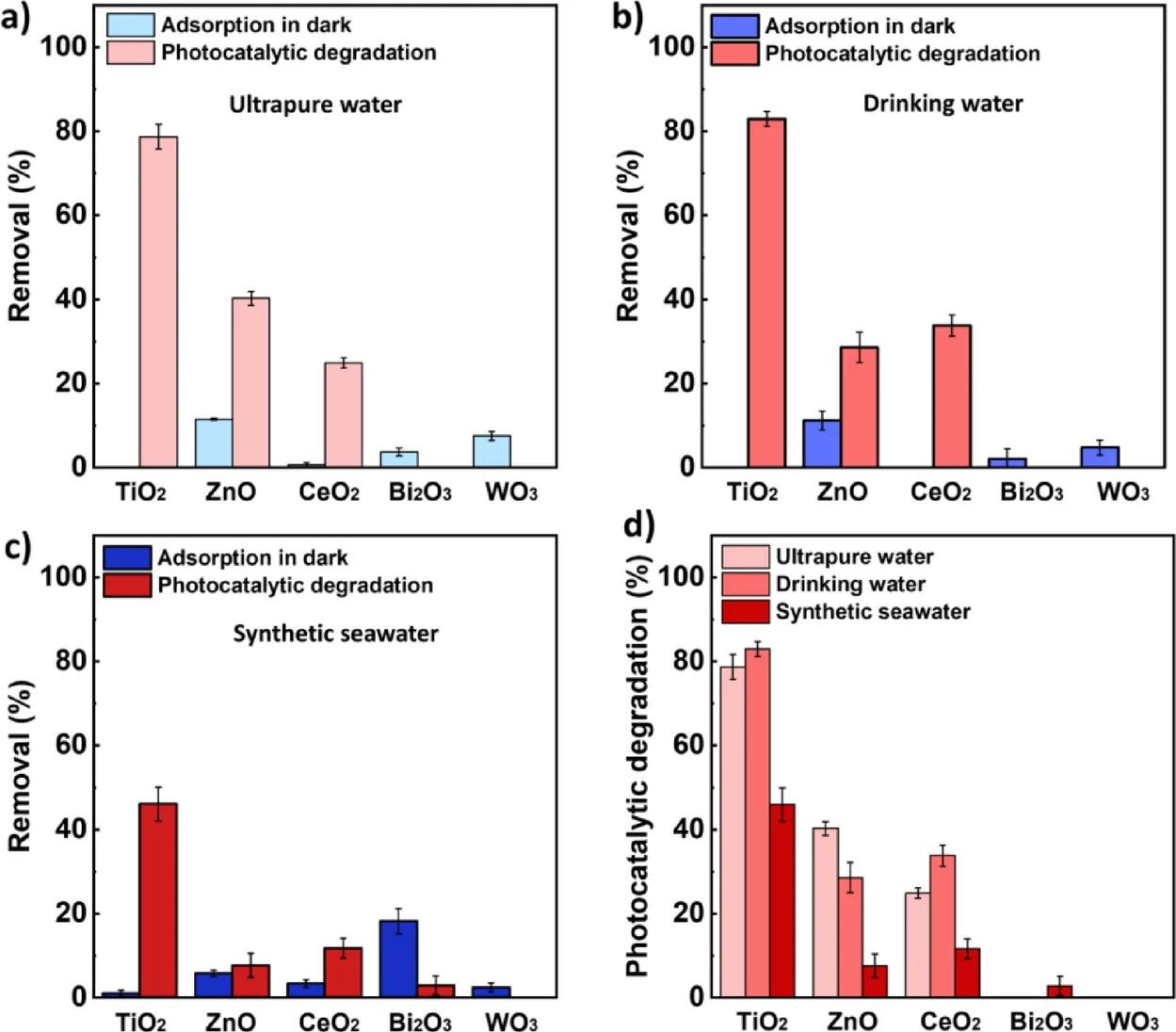
Adsorption efficiency in the dark (%) after 30 min and photocatalytic degradation efficiency (%) under UV radiation after 60 min of chloroquine phosphate (CLQ) (30 mg/L) by TiO_2_, ZnO, CeO_2_, Bi_2_O_3_ and WO_3_ nanoparticles in ultrapure water (**a**), drinking water (**b**), and synthetic seawater (**c**). Comparison of photocatalytic degradation efficiency (%) under UV radiation after 60 min of chloroquine phosphate (CLQ) (30 mg/L) in different water matrices (**d**).

The photocatalytic activity of the nanoparticles was evaluated for the degradation of chloroquine phosphate (CLQ) under UV radiation in different water matrices (Fig. 6; Table 4). Table 4 also presents some key parameters that influence the photocatalytic process. The photolysis and CLQ degradation under UV radiation in the presence of photocatalysts were performed in different water matrices (Figure S5) as a control experiment. No significant degradation of CLQ was observed in ultrapure water (Figure S5 a)), drinking water (Figure S5 b)) and synthetic seawater (Figure S5 c)) and the increased C/C_0_ efficiency (< 7%) was due to evaporation. The adsorption of CLQ in the dark (Figure S6 and Table S3) were also performed as a control experiment. All the nanoparticles presented negligible CLQ adsorption (< 10%) without irradiation across all water matrices, except for Bi_2_O_3,_ which showed nearly 20% adsorption in synthetic seawater. This high adsorption behaviour could be due to the surface interactions with CLQ and the influence of the synthetic seawater matrix composition on the adsorption process. Furthermore, the pH of the solution after 60 min of photocatalysis was measured, and there was no significant variation observed, suggesting relatively stable experimental conditions during the experiments.

- Ultrapure water

In ultrapure water (Fig. 6a), the photocatalytic activity of the nanoparticles followed the order: TiO_2_ > ZnO > CeO_2_. Among all samples, TiO_2_ exhibited the highest activity, reaching a CLQ removal efficiency of 79%, followed by ZnO and CeO_2_ with 40% and 25%, respectively. Additionally, neither Bi_2_O_3_ nor WO_3_ degraded CLQ. The high degradation performance of TiO_2_ can be attributed to its high surface area of TiO_2_, resulting from its small particle size, which promotes contact with CLQ molecules and thereby improves photocatalytic activity^86^. At the same time, TiO₂ was negatively charged in ultrapure water, while the CLQ molecule remained in its positively charged (cationic) form (pK_a_ = 8.4)^13^, enhancing electrostatic attraction between them. Additionally, the strong ROS-generating capacity of TiO_2_ also favoured the degradation of pollutants^27^. Compared to TiO₂, ZnO exhibited a much larger particle size, resulting in a lower specific surface area and possessed a higher DLS hydrodynamic diameter as a consequence of agglomeration in aqueous media, which limited its contact with CLQ. ZnO also showed a positively charged surface, which could repel electrostatically the positively charged CLQ, hindering the interaction with CLQ during photocatalysis. Furthermore, ZnO generated lower amounts of ROS than TiO_2_. Consequently, the degradation efficiency of ZnO for CLQ removal was lower than that of TiO_2_. Regarding CeO_2_, although it had a smaller particle size and DLS hydrodynamic diameter than TiO_2_ and ZnO, its interaction with CLQ was limited because CeO2 was positively charged, repelling CLQ electrostatically. Combined with its lower ROS-generation capacity compared to TiO₂ and ZnO, CeO₂ showed a reduced degradation efficiency. On the other hand, the null degradation efficiency of Bi_2_O_3_ and WO_3_ for CLQ removal was mainly due to their large particle sizes and hydrodynamic size, resulting in low surface areas and limited contact with pollutants, indicating reduced photocatalytic efficiency. Furthermore, Bi_2_O_3_ also generated the least ROS, thereby disfavouring CLQ degradation. WO_3_ was highly negatively charged during the reaction, implying the easy adsorption of the positively charged CLQ molecule on the WO_3_ particle’s surface by electrostatic interaction^27^. This adsorbed molecule could further reduce the active site and attenuate light absorption on the WO_3_ surface^86^ despite generating ROS similar to those of ZnO, leading to negligible photocatalytic activity.

- Drinking water

In drinking water (Fig. 6b), despite the presence of ions and a slightly acidic pH, the nanoparticles’ degradation behaviour could also be observed, following the order: TiO_2_ > CeO_2_ > ZnO. TiO_2_ exhibited the highest photocatalytic efficiency, degrading 83% of CLQ. CeO_2_ and ZnO degraded 34% and 29% of CLQ, respectively, under the same conditions. No photodegradation of CLQ was observed when Bi_2_O_3_ and WO_3_ were used as photocatalysts. As explained previously, the high degradation efficiency of TiO_2_ could be attributed to the high quantity of generated ROS and its high surface area^27,86^, despite its positively charged surface, which could disfavour the interaction with cationic CLQ molecules. Compared to TiO_2_, both ZnO and CeO_2_ showed highly positively charged surfaces, repelling CLQ molecules and decreasing the photocatalytic degradation of CLQ. However, CeO_2_ was more stable than ZnO in drinking water due to a high zeta potential value. Furthermore, acidic drinking water (pH = 5.3) increased ZnO dissolution as reported by Wu et al.^87^, thereby reducing its photocatalytic efficiency. Therefore, CeO_2_ degraded more CLQ than ZnO despite producing much lower ROS than ZnO. The deactivation of Bi_2_O_3_ for CLQ removal was mainly due to its inability to generate ROS in drinking water. In terms of WO_3_, its larger particle size and low ROS production limited its photocatalytic activity, leading to negligible CLQ degradation.

- Synthetic seawater

In synthetic seawater (Fig. 6c), despite the high ionic concentration and a slightly basic environment, the nanoparticles also exhibited degradation of CLQ, following the order TiO_2_ > CeO_2_ > ZnO. TiO₂ achieved a 46% CLQ degradation efficiency. This performance could be attributed to the negative surface charge of TiO₂, which promotes electrostatic attraction with cationic CLQ molecules, in combination with its high ROS-generating capacity and high surface area. ZnO tended to agglomerate in synthetic seawater due to its near-zero surface charge, limiting its contact with the CLQ molecule and resulting in low photocatalytic efficiency. The alkaline medium (pH = 8.2) of the synthetic seawater could increase the solubility of ZnO as reported by Leitner et al.^88^, further reducing its photocatalytic activity. In contrast to ZnO, CeO₂ exhibited a negatively charged surface under the same conditions, which may promote electrostatic attraction with cationic CLQ molecules. Although adsorption of positively charged ions in seawater may also occur simultaneously, the negatively charged surface of CeO_2_ facilitated interaction with CLQ molecules, thereby improving photocatalytic performance. As a result, ZnO achieved only 8% CLQ degradation, lower than the 12% obtained with CeO_2,_ despite both producing similar amounts of ROS. Additionally, Bi_2_O_3_ and WO_3_ degraded negligible amounts of CLQ because they were unable to generate ROS. In general, TiO_2_ exhibited high photocatalytic degradation of CLQ across all water matrices. The photocatalytic efficiency of ZnO and CeO_2_ was highly dependent on the water matrix. ZnO was more effective than CeO₂ in ultrapure water but was less effective in drinking water and synthetic seawater, where CeO₂ showed superior activity.

To further understand the influence of the water matrix on photocatalysis, a comparative analysis of CLQ degradation by the same nanoparticle in different water matrices was carried out, as shown in Fig. 6d. This analysis focused on TiO_2_, ZnO and CeO_2_ nanoparticles, which exhibited photocatalytic activity in degrading CLQ. TiO_2_ maintained the efficiency for CLQ degradation from ultrapure water to drinking water, but reduced efficiency by 50% in synthetic seawater, mainly due to reduced ROS generation. Furthermore, the presence of ions, especially in synthetic seawater, reduced the number of available active sites on TiO_2,_ thereby decreasing its photocatalytic activity. The photocatalytic efficiency of ZnO decreased by 30% in drinking water and up to 80% in synthetic seawater. This efficiency loss in ZnO may be attributed to pH variations in these water matrices compared to ultrapure water^43,44^, which promote ZnO dissolution and decrease efficiency^87–89^. Furthermore, the presence of ions also reduced ZnO’s photocatalytic efficiency, especially in synthetic seawater, where the high ionic concentration further blocked ZnO’s active sites. Additionally, the photocatalytic performance of CeO_2_ for CLQ removal was slightly enhanced in drinking water. This could be attributed to the stability of CeO_2_ in drinking water, its high zeta potential, and increased ROS generation. However, CeO_2_ showed a 50% decrease in photocatalytic activity in synthetic seawater compared to ultrapure water, despite a significant increase in ROS generation. This reduction could be explained by adsorbed ions on the CeO_2_ surface, which hinder the interaction between CeO_2_ and CLQ and compete with CLQ for active sites, thereby reducing the overall photocatalytic efficiency.

In general, the type of water matrix exerted a significant influence on the photocatalytic activity of TiO₂, ZnO, and CeO₂ for pollutant degradation. Moreover, the efficiency loss between ultrapure water and drinking water was not significant, whereas a considerable decrease in efficiency was observed in synthetic seawater. This significant difference in the efficiency reduction could be explained by the complex composition and high ion concentrations in synthetic seawater, which can compete for the active centre and attenuate the interaction between photocatalysts and pollutants^42,44^. Furthermore, the high conductivity of synthetic seawater can also hinder the adsorption of contaminants on the photocatalyst surface, thereby inhibiting photocatalytic activity^43,45^. Additionally, although the present work did not perform a mineralisation study of the contaminants, photocatalysis is a promising approach for CLQ removal, as previous studies have reported its effective mineralisation via photocatalysis^8,90^. Furthermore, it should be noted that the CLQ concentration used in this study is higher than environmentally relevant levels, such as 857 ng/L^12^. Nevertheless, the observed CLQ degradation performance implies the potential applicability of the TiO₂-based system for degrading lower concentrations typically found in real environmental conditions.

**Table 4:** Photocatalytic degradation efficiency (DE, %) of chloroquine phosphate (CLQ) under UV radiation after 60 min using TiO₂, ZnO, CeO₂, Bi₂O₃ and WO₃ nanoparticles in ultrapure water, drinking water, and synthetic seawater, along with the main factors (particle size, bandgap, Zeta-potential) that affect the photodegradation process.

Sample	Particle size (*≈*nm)	Bandgap - Eg (eV)	Ultrapure water (pH = 7)		Drinking water (pH = 5.3)		Synthetic seawater (pH = 8.2)	
			DE (%)	Zeta-potential (mV)	DE (%)	Zeta-potential (mV)	DE (%)	Zeta-potential (mV)
TiO_2_	27.9 ± 6.5	3.15 ± 0.03	79 ± 3	– 6.85 ± 0.31	83 ± 2	6.39 ± 0.10	46 ± 4	– 15.85 ± 0.07
ZnO	34.6 ± 13.5	3.07 ± 0.09	40 ± 2	28.25 ± 0.30	29 ± 4	34.88 ± 0.07	8 ± 3	2.60 ± 0.06
CeO_2_	18.1 ± 7.8	2.96 ± 0.06	25 ± 1	9.26 ± 0.41	34 ± 3	49.02 ± 0.06	12 ± 2	– 27.03 ± 0.26
Bi_2_O_3_	211.5 ± 92.7	2.27 ± 0.02	0 ± 0	9.70 ± 0.60	0 ± 0	31.60 ± 0.22	3 ± 2	– 5.99 ± 0.07
WO_3_	113.9 ± 38.3	2.50 ± 0.06	0 ± 0	– 50 ± 0.32	0 ± 0	– 28.93 ± 0.22	0 ± 0	– 48.40 ± 0.07

Although the conditions employed in the reported literature varied from work to work, and there is not a single study that evaluated and compared different photocatalysts against CLQ across different water matrices as the present work, Table 5 summarises the results from some relevant published works along with the applied experiments conditions. This type of comparison is not straightforward, but the comparative study allows contextualising of the results and novelty of our work.

As previously commented, the use of CLQ has increased during the COVID-19 pandemic^9,10^, and only a few works have reported its degradation by photocatalysis, especially across different types of water matrices. Concerning ultrapure water, our work shows results comparable to previous work, producing, in general, higher degradation efficiencies under UV radiation by TiO_2_ and ZnO. Compared to the experimental conditions used in our work, these results are expected, as previous works used much higher UV radiation intensities or lower initial CLQ concentrations. Furthermore, as highlighted previously, the degradation of CLQ and photocatalysis under real-world conditions remains limited. This limits direct comparison between our study and previously reported work. Therefore, our work addresses an important gap in the literature: the lack of comparative assessments of photocatalysts under realistic environmental conditions.

**Table 5:** Previous relevant works for chloroquine phosphate (CLQ) degradation under UV radiation.

Water matrix	CLQ (mg/L)	Type of Photocatalyst	Photocatalyst Concentration (mg/mL)	UV Radiation	Efficiency (%)	Time (min)	Ref.
Deionised water	20	TiO_2_,	0.1	UV 400 W	22	30	^8^
Deionised water	20	ZnO	0.1	UV 400 W	38	30	^8^
Ultrapure water	10	Bi_2_O_3_-TiO_2_	0.25	Xenon lamp with UV reflector 200–400 nm, 1500 W/m^2^ (300 W)	90	30	^46^
Ultrapure water	30	TiO_2_	1	3.3 W/m^2^ (8 W)	54	60	^27^
Ultrapure water	30	ZnO	1	3.3 W/m^2^ (8 W)	37	60	^27^

## Conclusion

Among all photocatalysts tested under UV irradiation, TiO₂ remained the outperformer, achieving the highest ROS yields and the most stable removal efficiencies across matrices. In drinking water, TiO₂ generated 601 µM·g⁻¹ of ^·^OH and 712 µM·g⁻¹ of ¹O₂, attaining 83% CLQ degradation. ZnO and CeO₂ showed intermediate performance, generating approximately 8 times less •OH and 5 times less its ¹O₂, indicating proportional decreases in ROS generation due to ion-induced surface passivation. Their photocatalytic performance became strongly matrix-dependent, with CLQ removal of 40 − 25% in ultrapure water and a significant reduction to 8–12% in synthetic seawater, confirming the suppression of both ROS and photocatalytic activity in more complex water matrices. Bi₂O₃ and WO₃ consistently produced low levels of ROS and showed no photocatalytic activity in any of the tested water matrices, suggesting that combining these two factors with complex water matrices results in low efficiency.

Overall, these findings demonstrate that photocatalyst ranking, typically presented in photocatalytic studies versus contaminants, is not intrinsic but matrix-dependent, and that evaluating materials solely in ultrapure water greatly overestimates their versatility and environmental applicability. By understanding how each semiconductor responds to specific water-matrix characteristics, this work provides a rare systematic assessment to facilitate the prediction of photocatalytic behaviour across diverse and complex water matrices, particularly for degrading CLQ and similar contaminants. The following steps will involve the immobilisation of nanoparticles into polymeric matrices and the evaluation of this system’s photocatalytic activity and reusability in real water matrices.

## Supplementary Information

Below is the link to the electronic supplementary material.

## Data Availability

Data will be made available on request.
