# Supplementary material for: Evaluation of metal-oxide semiconductors for the photocatalytic degradation of chloroquine phosphate in real-world water matrices
Source: Sci Rep. 2026 Jun 16;16:27620. doi: 10.1038/s41598-026-56732-x (PMC13538560; doi:10.1038/s41598-026-56732-x)
Supplement: Supplementary file 1 [file 41598_2026_56732_MOESM1_ESM.docx]

**Supplementary materials**

Evaluation of metal-oxide semiconductors for the photocatalytic degradation of chloroquine phosphate in real-world water matrices

Fangyuan Zheng ^a,b*^, Roberto Fernández de Luis ^c^, Senentxu Lanceros-Méndez ^c,d,e*^, Pedro M. Martins ^a,b^

^a^ Centre of Molecular and Environmental Biology (CBMA), University of Minho, 4710-057 Braga, Portugal

^b^ Institute for Research and Innovation on Bio-Sustainability (IB-S), University of Minho, 4710-057 Braga, Portugal

^c^ BCMaterials, Basque Center for Materials, Applications and Nanostructures, UPV/EHU Science Park, 48940 Leioa, Spain

^d^ Physics Centre of Minho and Porto Universities (CF-UM-UP) and LaPMET - Laboratory of Physics for Materials and Emergent Technologies, University of Minho, 4710-057 Braga, Portugal

^e^ IKERBASQUE, Basque Foundation for Science, 48009 Bilbao, Spain

**Corresponding author:* [fzheng@bio.uminho.pt](mailto:fzheng@bio.uminho.pt) (F. Zheng); [senentxu.lanceros@bcmaterials.net](mailto:senentxu.lanceros@bcmaterials.net) (S. Lanceros-Méndez)

**S-1: Chemical and physical properties of the commercial drinking water**

**S-2: Chemical and physical properties of the synthetic seawater**

**S-3: Protocol for the detection and quantification of Reactive Oxygen Species (ROS)**

**S-4: Size distribution of nanoparticles**

**S-5: Comparison of generated Reactive Oxygen Species (ROS) in different water matrices**

**S-6: Photolysis of chloroquine phosphate (CLQ) under UV radiation**

**S-7: Adsorption in the dark of chloroquine phosphate (CLQ)**

**S-1: Chemical and physical properties of the commercial drinking water**

**Table S 1.** Chemical and physical properties of the commercial drinking water.

| **Parameter** | **Value** |
| --- | --- |
| pH | 5.3 ± 0.4 |
| Temperature | 21 ºC |
| Conductivity | 55 µS/cm |
| Total mineralisation | 32 ± 2 mg/L |
| SiO_2_ | 9.0 ± 0.4 mg/L |
| Cl^-^ | 9.6 ± 0.4 mg/L |
| Na^+^ | 5.6 ± 0.4 mg/L |
| SO_4_^2-^ | 1.4 ± 0.2 mg/L |
| Mg^2+^ | 1.0 ± 0.2 mg/L |
| Ca^2+^ | 0.6 ± 0.2 mg/L |
| NO_3_^-^ | 1.9 ± 0.2 mg/L |
| HCO_3_^-^ | 2.9 ± 0.5 mg/L |

**S-2: Chemical and physical properties of the synthetic seawater**

**Table S 2.** Chemical and physical properties of the synthetic seawater.

| **Parameter** | **Value** |
| --- | --- |
| pH | 8.2 |
| Temperature | 21 ºC |
| Conductivity | 46.4 mS/cm |
| Salinity | 33.9 ‰ |
| Cl^-^ | 19.353 g/kg |
| Na^+^ | 10.765 g/kg |
| SO_4_^2-^ | 2.711 g/kg |
| Mg^2+^ | 1.295 g/kg |
| Ca^2+^ | 0.414 g/kg |
| K^+^ | 0.387 g/kg |
| HCO_3_^-^ | 0.142 g/kg |
| Br^-^ | 0.066 g/kg |
| Sr^2+^ | 0.008 g/kg |
| H_3_BO_3_ | 0.026 g/kg |
| F^-^ | 0.001 g/kg |

**S-3: Detection and quantification of Reactive Oxygen Species (ROS)**

**Figure S 1.** Hydroxylation reaction of terephthalic acid (TA).

**Figure S 2.** Mechanism of the histidine test.

**S-4: Size distribution of nanoparticles**

**Figure S 3.** Size distribution obtained after measuring 100 nanoparticles by Image J for TiO_2_ (a), ZnO (b), CeO_2_ (c), Bi_2_O_3_ (d) and WO_3_ (e) nanoparticles.

**S-5: Comparison of generated Reactive Oxygen Species (ROS) in different water matrices**

**Figure S 4**. Comparison of photogenerated hydroxyl radical (•OH) (a) and singlet oxygen (^1^O_2_) (b) by TiO_2_, ZnO, CeO_2_, Bi_2_O_3_ and WO_3_ nanoparticles in different water matrices under 60 min of UV radiation.

**S-6: Photolysis of chloroquine phosphate (CLQ) in different water matrices under UV radiation**

**Figure S 5.** Photolysis assay of CLQ (30 mg/L) under 60 minutes of UV radiation in ultrapure water (a), drinking water (b) and synthetic seawater (c).

**S-7: Adsorption in the dark of chloroquine phosphate (CLQ) in different water matrices**

**Figure S 6.** Comparison of adsorption efficiency (%) of CLQ (30 mg/L) in the dark after 30 min using TiO_2_, ZnO, CeO_2_, Bi_2_O_3_ and WO_3_ in different water matrices.

**Table S 3.** CLQ adsorption efficiency (AE, %) in the dark after 30 min by TiO_2_, ZnO, CeO_2_, Bi_2_O_3_ and WO_3_ nanoparticles in ultrapure water, drinking water and synthetic seawater.

| Sample | Adsorption efficiency (AE, %) in the dark | | |
| --- | --- | --- | --- |
|  | Ultrapure water | Drinking water | Synthetic seawater |
| TiO_2_ | 1 ± 0 | 1 ± 4 | 1 ± 1 |
| ZnO | 11 ± 0 | 11 ± 2 | 6 ± 1 |
| CeO_2_ | 1 ± 1 | 1 ± 1 | 3 ± 1 |
| Bi_2_O_3_ | 4 ± 1 | 2 ± 2 | 18 ± 3 |
| WO_3_ | 8 ± 1 | 5 ± 2 | 2 ± 1 |
